# Verification and Defined Dosage of Sodium Pentobarbital for a Urodynamic Study in the Possibility of Survival Experiments in Female Rat

**DOI:** 10.1155/2020/6109497

**Published:** 2020-06-11

**Authors:** Sheng-Fei Xu, Guang-Hui Du, Kuerbanjiang Abulikim, Peng Cao, Hui-bing Tan

**Affiliations:** ^1^Department of Urology, Tongji Hospital, Tongji Medical College, Huazhong University of Science and Technology, Wuhan, Hubei Province, China; ^2^Department of Anatomy, Jinzhou Medical University, Jinzhou, Liaoning Province, China

## Abstract

**Objectives:**

To evaluate the effects of pentobarbital dosages on lower urinary tract function and to define an appropriate dosage of sodium pentobarbital that would be suitable for urodynamic studies in which recovery from anesthesia and long term survive were needed for subsequent experiment.

**Methods:**

Twenty-four 8-week-old, female, virgin, Sprague-Dawley rats (200-250 g) were used in this study. Rats in study groups received gradient doses of pentobarbital intraperitoneally, and those in the control group received urethane intraperitoneally. External urethral sphincter electromyography (EUS-EMG) was recorded simultaneously during cystometry and leak point pressure tests. The toe-pinch reflex was used to determine the level of anesthesia.

**Results:**

Micturition was normally induced in both the urethane group and 32 mg/kg pentobarbital group. However, in groups of 40 mg/kg or 36 mg/kg pentobarbital, micturition failed to be induced; instead, nonvoiding contractions accompanied by EUS-EMG tonic activity were observed. There were no significant differences in leak point pressure or EUS-EMG amplitude or frequency between the urethane and 32 mg/kg pentobarbital groups.

**Conclusions:**

This study confirmed significant dose-dependent effects of pentobarbital on lower urinary tract function and 32 mg/kg pentobarbital as an appropriate dosage for recovery urodynamic testing, which enable the achievement of expected essential micturition under satisfactory anesthesia in female rats.

## 1. Introduction

Anesthesia is a necessary procedure for most urodynamic studies of lab animals, although the fully awake animals have been assessed in some urodynamic tests [1, 2]. The optimal anesthetic procedure is the one that results in minimal impact on urodynamic parameters while providing satisfactory anesthetic conditions. Additionally, some urodynamic studies are necessarily performed in the awake animals of recovery from anesthesia and survive for a certain period of time for subsequent experiments [3]. Afferent neurotransmission from the periphery to supraspinal sensory centers is unaffected by urethane anesthesia. Therefore, urethane has become accepted as a preferred anesthetic for urodynamic studies due to its mild effects on the micturition in rats [4, 5]. However, due to its potential toxicity to both investigators and lab animals, the use of urethane is under strict regulation in some countries, and it is not recommended for recovery experiments due to high mortality after anesthesia; thus, it is only suitable for nonsurvival experiments in rats [6–10].

Sodium pentobarbital, a short-acting GABA_A_-receptor potentiator [11], is a reliable anesthetic for survival experiments, which is often used in urodynamic studies [4, 9, 12–28]. However, pentobarbital can abolish the micturition in some cases [12, 29, 30]. In 1986, Yaksh and colleagues reported that the administration of sodium pentobarbital produced a complete blockade of the volume-evoked micturition reflex [12]. In 1987, Malmgren et al. also reported that no micturition occurred during cystometry conducted during pentobarbital anesthesia [29]. In the above studies, the dosage of sodium pentobarbital was 50 mg/kg. By referring to the literature about anesthesia and surgery of laboratory animals, we noted that the suggested dosage of sodium pentobarbital for intraperitoneal injection was 30-40 mg/kg in rats [31, 32]. There were remarkable differences in the dosages of pentobarbital between the recommended dosage and the dosage used in those studies reporting micturition blockage.

The previous studies have shown that the pentobarbital produced a dose-dependent inhibition of heart rate, blood pressure, and respiration [33, 34]. Similarly, the GABA receptor agonists produced a dose-dependent inhibition of micturition, with micturition inhibited only in high doses [35]. Although it has been well known that high-dose pentobarbital depresses micturition in rats [12, 29], up to now, the detail and precise effects of low-dose pentobarbital (40 mg/kg or less) on micturition remain unclear in rats. To answer these questions and to define an appropriate dosage of sodium pentobarbital for urodynamic studies, we conducted the present study in female rats to investigate the dose-dependent effect of pentobarbital on lower urinary tract function, with urethane anesthesia as a positive control.

## 2. Materials and Methods

### 2.1. Ethics Statement

All procedures involving animals were conducted in accordance with the guidelines of the Chinese Council on Animal Care and with approval from the Committees on Animal Experiments at Tongji Hospital (Tongji Medical College, Huazhong University of Science and Technology, Wuhan, China). All rats were housed in separated cages in a temperature-controlled room with a 12 h light/dark cycle and free access to food and water. All surgeries were performed under anesthesia, and all efforts were made to minimize animal suffering.

### 2.2. Animals and Experimental Design

Twenty-four 8-week-old, female, virgin, Sprague-Dawley rats (200-250 g) were used in this study. Rats were divided into 4 groups: the control group received urethane (1.2 g/kg) intraperitoneally (i.p., *n* = 6) [36]; the other 3 groups received graded doses of sodium pentobarbital intraperitoneally at 32 (*n* = 6), 36 (*n* = 6), and 40 (*n* = 6) mg/kg. The toe-pinch reflex was used to determine the level of anesthesia in the present study [37].

### 2.3. Surgical Preparation

Anesthesia was induced with 3.5% isoflurane in oxygen, and surgery was carried out at 2.5% isoflurane in oxygen. A midline abdominal incision was made to expose the bladder under a microscope. A 15 cm length polyethylene catheter (PE-50) with a flared tip (inner diameter: 0.58 mm, outer diameter: 0.96 mm) was inserted into the bladder dome to measure intravesical pressure of the bladder, and a purse-string suture was used to close the bladder dome incision tightly. The rest of the catheter was tunneled subcutaneously and secured with a hitch suture to the rectus fascia and lower abdominal skin to prevent slippage. The pubic symphysis and the medial portion of the pubic bones were removed to expose the mid portion of the urethra. When preparatory surgery was completed, the isoflurane anesthesia was turned off. Thereafter, an intraperitoneal injection of sodium pentobarbital or urethane was administered for subsequent urodynamic measurements.

### 2.4. Filling Cystometry with Simultaneous External Urethral Sphincter Electromyography (EUS-EMG) Recordings

**Figure 1:**
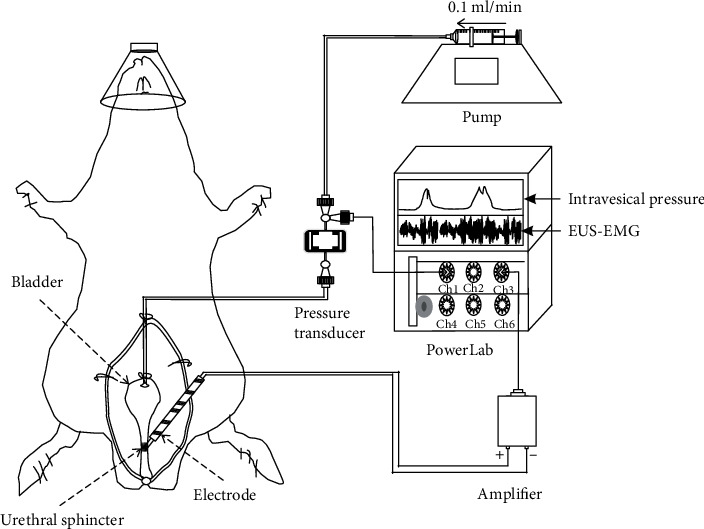
Schematic of cystometry with simultaneous external urethral sphincter electromyography recording in rat. EUS-EMG: external urethral sphincter electromyography; Ch: channel.

For cystometry, the opposite end of the bladder catheter was attached to a 3-way connector connected to both a programmable infusion pump and a pressure transducer to support bladder infusion and bladder pressure recordings, respectively. For recording EUS-EMG activity, a parallel bipolar electrode was placed on the surface of the EUS bilaterally at the midurethra. CMG and EUS-EMG data were amplified and digitized using a multiple channel electrophysiological recording system (PowerLab 26T, AD Instruments, Australia). The perfusion speed was set to 0.1 ml/min (Figure 1). The cystometry signal was recorded at 1 kHz and low-pass filtered at 100 HZ, and the EMG signal was recorded at 6 kHz with band pass frequencies from 20 Hz to 3 kHz. The segment of the EMG was filtered for 60 Hz noise (power supply frequency). A total of 3-4 consecutive voiding cycles were collected. The intercontraction interval (ICI), pressure threshold (PT), resting pressure (RP), maximum intravesical pressure (MIVP), contraction duration (CD), expulsion time (ET), and bursting activity period (BP) were determined according to established criteria [38, 39].

### 2.5. Leak Point Pressure (LPP) with Simultaneous EUS-EMG Recordings

LPP recordings were performed after cystometry determinations. For LPP testing, the bladder was filled to half capacity [40]. An external increase in bladder pressure was made by slowly pressing on the bladder using a cotton swab and removing it quickly at the first sign of fluid leakage at the urethral meatus. A mean of 4 LPP tests was performed in each animal, and a mean of the results was calculated and used. LPP was calculated as baseline pressure subtracted from peak pressure [41]. EUS-EMG activity at baseline and at the peak pressure of the LPP test was analyzed in three or four 1-second samples, as previously described [36].

### 2.6. Statistical Analysis

The values for each parameter were averaged for each rat, and the quantitative data were expressed as the means ± standard error (SE). EUS-EMG analysis using Matlab software. Data of EUS-EMG and LPP were compared using one-way analysis of variance (ANOVA). The filling cystometry data obtained in the urethane- and 32 mg/kg pentobarbital-anesthetized groups were compared statistically using Student's *t*-test. We regarded a *P* value of less than 0.05 to indicate a statistically significant difference between groups.

## 3. Results

### 3.1. Micturition

**Figure 2:**
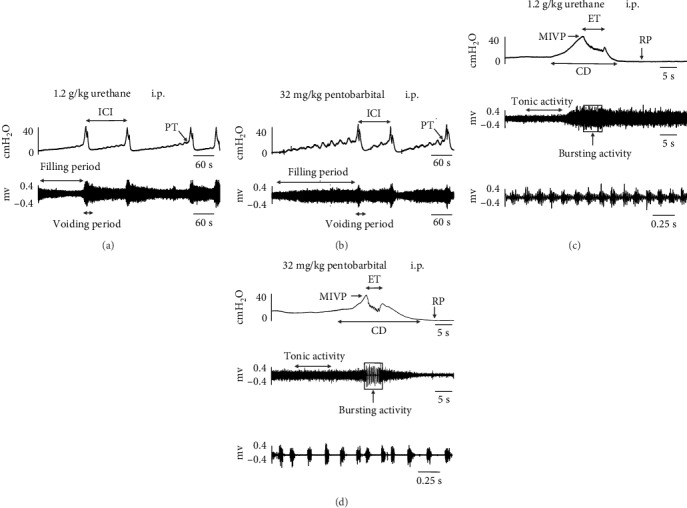
Typical examples of cystometry under anesthesia with urethane and 32 mg/kg sodium pentobarbital. Micturition could be observed in the urethane group (a) and 32 mg/kg pentobarbital group (b). (c) and (d) shows tonic EUS-EMG activity shifted to a bursting pattern at the peak of the bladder contraction in the urethane and 32 mg/kg pentobarbital groups. Top, cystometrogram; middle, EUS-EMG activity during filling and voiding; bottom, EUS bursting activity during voiding in faster time phase. EUS-EMG: external urethral sphincter electromyography; ICI: intercontraction interval; PT: pressure threshold; RP: resting pressure; MIVP: maximum intravesical pressure; CD: contraction duration; ET: expulsion time; i.p.: intraperitoneal injection.

**Figure 3:**
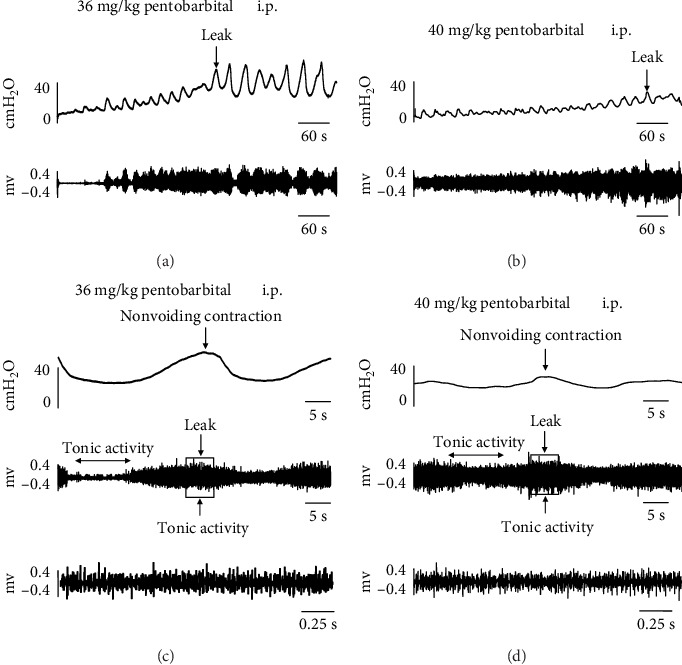
Typical examples of cystometry under anesthesia with 36 and 40 mg/kg sodium pentobarbital. Micturition could not be observed in the 36 mg/kg group (a) and 40 mg/kg pentobarbital group (b). (c) and (d) shows increased tonic EUS-EMG activity during a nonvoiding contraction with a bladder leak response in the 36 mg/kg and 40 mg/kg pentobarbital groups. Top, cystometrogram; middle, EUS-EMG activity during filling and nonvoiding contraction; bottom, EUS bursting activity during nonvoiding contraction in faster time phase. EUS-EMG: external urethral sphincter electromyography; i.p.: intraperitoneal injection.

In all rats that received 32 mg/kg pentobarbital and urethane anesthesia, the micturition could be observed, and all rats could be kept quiet during bladder filling (Figure 2). However, in all rats anesthetized with 40 mg/kg and 36 mg/kg pentobarbital, no micturition could be evoked instead of nonvoiding contractions, with all rats keeping quiet during bladder filling (Figure 3). The mean duration of the loss of the micturition was a significant difference between the control group and the group of 32 mg/kg pentobarbital anesthesia, which amounted to 51.67 ± 4.63 and 78.75 ± 4.15 minutes, respectively (*p* < 0.05). However, in rats that received 40 and 36 mg/kg pentobarbital, micturition was suppressed for more than 3 hours. The toe-pinch reflex was negative in all study groups.

### 3.2. EUS-EMG

In all rats that received 32 mg/kg pentobarbital and urethane anesthesia, the tonic EUS-EMG activity slowly increased during bladder filling and closely matched the rise in intravesical pressure at the onset of bladder contractions. During the voiding phase, the EUS-EMG changed from tonic activity to bursting activity. After voiding, the intravesical pressure returned to baseline, and the bursting EUS-EMG activity shifted to a tonic pattern again (Figure 2).

In rats that received 40 and 36 mg/kg pentobarbital, the micturition disappeared and was replaced by nonvoiding contractions. Each nonvoiding contraction corresponded to increased tonic activity of EUS-EMG. Leakage occurred subsequent to bladder filling and was accompanied by increased tonic activity of the external urethral sphincter, but large-amplitude EUS-EMG bursting activity and large-amplitude reflex bladder contractions were eliminated (Figure 3).

### 3.3. Cystometric Parameters

**Figure 4:**
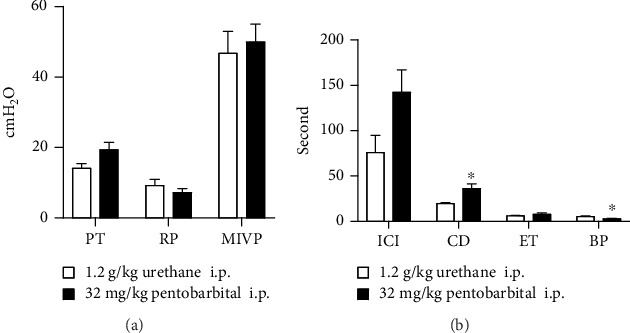
Parameters of cystometry results under anesthesia with urethane and 32 mg/kg sodium pentobarbital. Data reported as the mean ± SEM. PT: pressure threshold; RP: resting pressure; MIVP: maximum intravesical pressure; ICI: intercontraction interval; CD: contraction duration; ET: expulsion time; BP: bursting activity period; i.p.: intraperitoneal injection. ^∗^*p* < 0.05.

For a more detailed assessment of the two anesthetic agents on the contractile properties of the bladder, we compared the parameters of cystometry in rats that received 32 mg/kg pentobarbital anesthesia with those in rats of the urethane group. There were significant differences in CD and BP between the two groups. However, no significant differences in ICI, PT, RP, MIVP, and ET were observed (Figure 4).

### 3.4. LPP Testing

**Figure 5:**
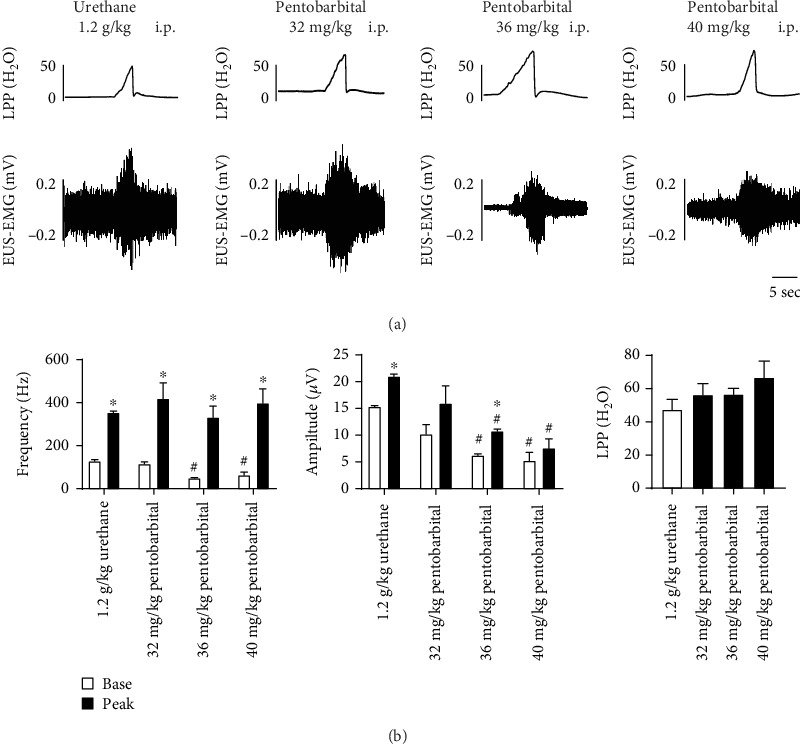
LPP and EUS-EMG results under anesthesia with urethane and different dosages of pentobarbital. Data reported as the mean ± SEM. Representative examples of EUS-EMG activity indifferent anesthesia groups (a). Top, LPP; bottom, EUS-EMG activity during LPP. ∗ indicates a statistically significant difference compared to data at baseline in the same group. # indicates a statistically significant difference compared to rats that had anesthesia with urethane (*p* < 0.05). LPP: leak point pressure; EUS-EMG: external urethral sphincter electromyography; i.p.: intraperitoneal injection.

No significant differences in LPP were observed among the four groups. In all rats that received 32 mg/kg pentobarbital and urethane anesthesia, no significant differences in EUS-EMG frequency and amplitude were observed (Figure 5).

## 4. Discussion

It is well known that GABA can inhibit reflex-activated bladder motility by acting though different distinct sites [35]. Sodium pentobarbital, a short-acting GABA_A_-receptor potentiator [11], enhances inhibition mediated by GABA_A_, increasing the average burst duration of activated GABA_A_ receptor channels [42], which may be one of the mechanisms of pentobarbital inhibiting lower urinary tract function. In a previous study, the doses of sodium pentobarbital intraperitoneal injection were administrated from 30 mg/kg to 60 mg/kg of body weight in rat for urodynamic experiments [12, 17, 23, 26, 28, 43]. However, high dose inhibits micturition and causes higher mortality for recovery experiments [12, 29, 30]. In the present study, we defined the precise effects of gradient dosages of sodium pentobarbital on micturition. In rats that received 40 mg/kg and 36 mg/kg sodium pentobarbital, the micturition could not be maintained, which is consistent with previous studies [12, 29], while in rats that received 32 mg/kg pentobarbital, the micturition could be induced in all rats. Additionally, the toe-pinch reflex was negative in all groups of rats during urodynamic recording, which is indicative of a satisfactory level of anesthesia.

In 2000, Matsuura and Downie [4] reported that the micturition was suppressed under pentobarbital at 30-50 mg/kg (i.p.). The possible explanation is that there were two questionable debates in the experimental method reported by Matsuura and Downie [4], which led to their results being inaccurate. First, their study only described that the dose of sodium pentobarbital in the experiment was 30-50 mg/kg, but the study lacked detailed evaluation of the effect of different doses of sodium pentobarbital on micturition. Second, the sample size of the study was small and only 4 rats were studied. From experimental methodology, we found that animals were not studied in groups. Therefore, the results of this experiment cannot conclude that intraperitoneal injection of sodium pentobarbital at low dose (40 mg/kg or less) can inhibit micturition. However, to our knowledge, we are the first to use serial decrement doses of sodium pentobarbital to evaluate the effects of low-dose pentobarbital (40 mg/kg or less) on micturition in female rats. Our evidence showed that, among these doses of sodium pentobarbital, only 32 mg/kg sodium pentobarbital can sparely be available for the micturition. Additionally, in our recent studies, it was shown that micturition was suppressed for 78.75 ± 4.15 minutes after anesthesia with 32 mg/kg pentobarbital, which is in line with a previous study [9]. The study showed that pentobarbital can cause the micturition to disappear for a long time (60-90 minutes). However, there was an anesthesia window allowing for micturition reflex studies for more than an hour [9].

In the present study, our results still suggested that the ICI in the pentobarbital group (32 mg/kg) was longer than that in the urethane group, although no significant difference was observed. Such prolongation of the ICI may imply an increased bladder capacity [39]. Furthermore, a shortened BP in the 32 mg/kg pentobarbital group was observed compared with that in the urethane group. The shorter BP may reduce efficient bladder emptying, although a longer CD was observed compared with the urethane group. This effect of anesthetic on cystometry should be taken into consideration when cystometry is anesthetized with pentobarbital at a dosage of 32 mg/kg.

Previous studies reported that EUS bursting is a critical event and necessary for efficient bladder emptying [44, 45]. When 40 mg/kg and 36 mg/kg pentobarbital were administered, the external urethral sphincter showed an increased tonic activity without a large-amplitude EUS-EMG bursting activity, which was similar with detrusor sphincter dyssynergia, and cannot produce efficient voiding. However, in the 32 mg/kg pentobarbital, the EUS-EMG changes from tonic activity to bursting activity during the voiding phase. After voiding, the bursting EUS-EMG activity shifts to a tonic pattern again. These characteristics of EMG are similar with those with urethane anesthesia.

The bladder-to-urethral reflex has been proved to be an important role in the prevention of urinary leakage [46]. When a passive external pressure is applied to the bladder (LPP), the EUS-EMG activity is increased to elevate the urethral resistance. In the present study, rats that received 40 mg/kg, 36 mg/kg, and 32 mg/kg sodium pentobarbital demonstrated typical LPP and EUS-EMG data with a statistically significant increase in EUS-EMG frequency during LPP testing, considering that the bladder-to-urethral reflex also exists in female rats by pentobarbital anesthesia. In the present study, compared with urethane anesthesia, LPP was not significantly different after anesthesia with 40 mg/kg, 36 mg/kg, and 32 mg/kg sodium pentobarbital.

## 5. Study Limitations

Although this study found that micturition was not suppressed when rats are anesthetized with low-dose sodium pentobarbital, the present study has several limitations. First, the study groups do not include a lower dosage of pentobarbital than 32 mg/kg, due to the fact that the animal could not get quite sufficient when the dosage of pentobarbital decreased lower than 32 mg/kg. Second, the mechanism of pentobarbital sodium on micturition reflex has not been studied, mainly hampered by our knowledge scope and lab facilities, for example, pharmacological study and signaling transduction of GABA_A_ receptor channels. Third, our study only observed the effect of pentobarbital sodium on micturition in female rats, and the dose-dependent effects of pentobarbital on lower urinary tract function in other species need to be investigated in the future. Fourth, we used urethane as an anesthetic agent for the urodynamic study in the control group; it may be more convincing had the sodium pentobarbital group be compared with the urodynamic data in the awake animals.

## 6. Conclusions

Pentobarbital was used to be considered to have obvious influence on lower urinary tract function. Thus, pentobarbital is not recommended for urodynamic experiments under a previous protocol. In the present study, we established a survival model for examining external urethral sphincter electromyography simultaneously during testing cystometry and leak point pressure tests by means of defining the dosage-dependent effects of pentobarbital on the lower urinary tract function and provide a nonurethane protocol, a suitable and practicable dosage of pentobarbital anesthesia in recovery urodynamic experiments with female rats. We found that high pentobarbital doses remarkably suppress lower urinary tract function, with impaired bladder contractions and EUS-EMG activation. Under a critical low-dose pentobarbital anesthesia, the lower urinary tract function is less affected, with analogous bladder contraction and EUS-EMG activation patterns comparable with that of urethane anesthesia.

In summary, this study confirmed significant dose-dependent effects of pentobarbital on lower urinary tract function and 32 mg/kg pentobarbital as an appropriate dosage for recovery urodynamic testing, which enable the achievement of expected essential micturition under satisfactory anesthesia in female rats.

## Data Availability

The data used to support the findings of this study are included within the article.
